# Facial Cosmetics and Attractiveness: Comparing the Effect Sizes of Professionally-Applied Cosmetics and Identity

**DOI:** 10.1371/journal.pone.0164218

**Published:** 2016-10-11

**Authors:** Alex L. Jones, Robin S. S. Kramer

**Affiliations:** 1 Department of Psychology, Swansea University, Swansea, United Kingdom; 2 Department of Psychology, University of York, York, United Kingdom; Tokyo Daigaku, JAPAN

## Abstract

Forms of body decoration exist in all human cultures. However, in Western societies, women are more likely to engage in appearance modification, especially through the use of facial cosmetics. How effective are cosmetics at altering attractiveness? Previous research has hinted that the effect is not large, especially when compared to the variation in attractiveness observed between individuals due to differences in identity. In order to build a fuller understanding of how cosmetics and identity affect attractiveness, here we examine how professionally-applied cosmetics alter attractiveness and compare this effect with the variation in attractiveness observed between individuals. In Study 1, 33 YouTube models were rated for attractiveness before and after the application of professionally-applied cosmetics. Cosmetics explained a larger proportion of the variation in attractiveness compared with previous studies, but this effect remained smaller than variation caused by differences in attractiveness between individuals. Study 2 replicated the results of the first study with a sample of 45 supermodels, with the aim of examining the effect of cosmetics in a sample of faces with low variation in attractiveness between individuals. While the effect size of cosmetics was generally large, between-person variability due to identity remained larger. Both studies also found interactions between cosmetics and identity–more attractive models received smaller increases when cosmetics were worn. Overall, we show that professionally-applied cosmetics produce a larger effect than self-applied cosmetics, an important theoretical consideration for the field. However, the effect of individual differences in facial appearance is ultimately more important in perceptions of attractiveness.

## Introduction

Modification of the body with dyes, paints, and other pigments is among the most universal of human behaviours, present in all cultures [1–3]. However, in Western society, women perform the majority of self-adornment [4], and perhaps the most prevalent behaviour of this kind is the use of facial cosmetics. This behaviour is served by the global cosmetics industry which is worth billions of pounds [5].

Women report using cosmetics for a variety of reasons, ranging from anxiety about facial appearance, conformity to social norms, and public self-consciousness [6–8], through to appearing more sociable and assertive to others [6]. Cosmetics are effective at improving social perceptions that the wearer may wish to modulate, with individuals appearing to be healthier and earning more [9], displaying greater competence, likeability and trustworthiness [10], as well as appearing more prestigious and dominant [11]. Cosmetics also influence the behaviour of others, especially men, who tip higher amounts and with greater frequency to waitresses wearing cosmetics [12], and are more likely to approach wearers in the environment [13]. It is likely that the effect of cosmetics on social perceptions is brought about by the increase in attractiveness it confers to faces, which is now a well documented effect [10,14–17]. Research has documented cosmetics function by altering sex-typical colouration in faces such as facial contrast [18–21], by increasing the homogeneity of facial skin [22,23], or by affecting colour cues to traits such as health [24] and age [25].

While the effect of cosmetics on perceived attractiveness seems clear [14,17], other research has revealed it is more nuanced than previously thought. Etcoff and colleagues [10] demonstrated that attractiveness increased linearly with the amount of cosmetics worn—simply, more cosmetics equates to appearing more attractive. Of the range of cosmetics that can be worn, the quantity of cosmetics applied to the eyes and mouth have been shown to be significant predictors of attractiveness [26], with more cosmetics on these features leading to higher ratings of attractiveness. However, other evidence suggests that the typical amount of cosmetics applied by a sample of young women is excessive, with observers preferring close to half the actual amount for optimal attractiveness [16], calling into question the linear relationship between cosmetics quantity and attractiveness.

One concern of facial attractiveness research is that it does not compare the effects of predictors of attractiveness (e.g., symmetry, averageness, sex typicality [27]; against other sources of variation [28]. Recent work has begun to address this by examining the importance of within-person variation in attractiveness (caused by the presence or absence of makeup, for example), compared with the between-person variation in attractiveness simply due to differences between identities [29]. Specifically, it has been previously shown that the effect of cosmetics on attractiveness, a source of within-person variation, is very small, explaining just 2% of the variance in ratings [15]. This is an especially small effect when compared with differences in attractiveness between individuals, a between-person variation in attractiveness, which explained 69% of the variance in judgements. More simply, while facial cosmetics do increase attractiveness, that contribution is small and does little to change an individual’s attractiveness standing in the population.

However, the use of cosmetics is an idiosyncratic and extremely varied practice [3], and its effect on attractiveness is more complex than previously thought. The use of a professional makeup artist is a common practice in almost all studies examining the effect of cosmetics on perceptions [9,10,12,17,30,31], and only a few utilise self-applied cosmetics [14,16,26]. An initial examination of the effect size of cosmetics on attractiveness also had models self-apply their cosmetics [15]. There are good reasons for using professionally-applied cosmetics, as it provides a clearer test of how cosmetics alter facial attractiveness. The increased variability in self-applied cosmetics, due, for example, to differences in application skill or the products used, could make it more difficult to detect an effect of cosmetics on attractiveness, and previous work has indeed found the effect to be small [15]. This distinction represents a trade-off between experimental control and ecological validity—the vast majority of women, if any, do not have a professional makeup artist apply their cosmetics daily, yet the majority of studies examining cosmetics and attractiveness draw conclusions based on professionally-applied cosmetics, which may only indirectly inform as to how cosmetics affect attractiveness in the real world.

We seek to address important theoretical points regarding how cosmetics influence attractiveness. How large is the effect size of cosmetics on attractiveness when cosmetics have been professionally-applied? If cosmetics in psychological experiments are applied with more skill than is typically achieved, then current knowledge of cosmetics and attractiveness likely overstates the relationship, given the reliance on professionally-applied cosmetics in the literature. Moreover, how does the ability of professionally-applied cosmetics compare to previous measures of the effect of cosmetics on attractiveness? In the following study, we examine the effect size of cosmetics on attractiveness in two sets of faces that have had cosmetics applied professionally, with the prediction that the effect will be substantially larger than the previous assessment that considered self-applied cosmetics [15]. In addition, by using a similar design to previous research, we can draw direct comparisons with current knowledge of how cosmetics and identity affect attractiveness.

A separate but related question regarding cosmetics concerns how it affects faces of different levels of attractiveness. Many studies in the literature on cosmetics and social perceptions have used models recruited from university or college [14,15,20]. How do cosmetics affect faces of a different population, specifically faces considered to be very attractive? Previous research found no interaction between cosmetics and identity [15], suggesting cosmetics affect each face’s attractiveness similarly. However, the models used were of a university-aged sample of population-typical attractiveness levels. The present studies, particularly Study 2, examine the effect cosmetics have on perceived attractiveness in a sample of women typically considered to be very attractive—models. Using a sample of faces that are already constrained in attractiveness enables us to manipulate another source of variation in attractiveness, specifically between-person variability. As such, we can observe the effects of cosmetics on attractiveness in a sample with a (hypothesised) lower effect of identity (differences between individuals) than elsewhere.

The present study has several aims. First, we examine how cosmetics affect attractiveness when cosmetics have been professionally-applied. We predict that cosmetics will have a notably larger effect size in this sample compared to the previous study examining this question [15]. Second, we consider the effect size of cosmetics in sets of faces that are considered highly attractive, where between-person variation (identity effect size) should be reduced. The relative effect size of cosmetics may therefore be increased, and may be more likely to overshadow the smaller between-person variation in attractiveness. Conversely, cosmetics may have less of an effect in these samples as the women are already at the higher end of attractiveness without cosmetics, leaving little room for judgements of attractiveness to increase when cosmetics are applied. Finally, by using an identical design to previous research [15], we will compare the findings obtained in these studies to those presented in previous research in order to build a fuller picture of the relative importance of cosmetics and identity in attractiveness perceptions.

## Study 1

In the first study, we examine how cosmetics impact attractiveness when they are applied professionally. To do this, we take advantage of an Internet-based sample to acquire images of models whose cosmetics have been applied by high-profile makeup artists. Compared to previous work examining this question [15], we predict that the effect size due to cosmetics should be larger here. However, the effect size of identity may still overshadow it.

### Method

#### Participants

Ninety North American university students (age *M* = 18.57 years, *SD* = 0.75, 41 men) participated in the main study for course credit. Due to a software error, age data was not recorded for the first 50 participants, with the mean age being calculated from the remaining participants. However, all participants were within the same demographic and age range. A further 15 students (age *M* = 19.93 years, *SD* = 1.16, three men) rated the quantity of cosmetics worn by the models. Informed consent was obtained from all participants included in the study.

#### Ethics Statement

Ethical approval for all studies was obtained from the Gettysburg College institutional review board (IRB). All participants gave written informed consent before beginning the study.

#### Stimuli

From the YouTube website, we collected images of White British women (*n* = 33, age unknown but approximately 20–35 years), who acted as models while their cosmetics were applied by high-profile professional makeup artists from the United Kingdom. Twenty-three models were obtained from one artist’s channel (www.youtube.com/user/lisaeldridgedotcom) with a further ten collected from another (www.youtube.com/user/ctilburymakeup). We utilised all available videos at the time of writing that featured a model receiving a makeover where they were shown before and after an application of cosmetics. In addition, we included only videos where faces began free of cosmetics, and the artist had the intention of applying a particular cosmetics look, rather than with the aim of hiding blemishes or skin conditions (such as acne). Images were captured from video tutorials, which served to instruct viewers on a number of popular cosmetics styles for a range of scenarios. Both authors classified the cosmetics looks into categories using information provided by descriptions within the videos. Three categories were apparent—an everyday, natural look (*n* = 7), a ‘going out’ look (*n* = 14), and vintage or editorial looks based on cosmetics the makeup artist had applied during professional photo shoots in the past (*n* = 12). A third researcher, with extensive experience in this field, arrived at these three categories independently, providing further confirmation.

**Fig 1:**
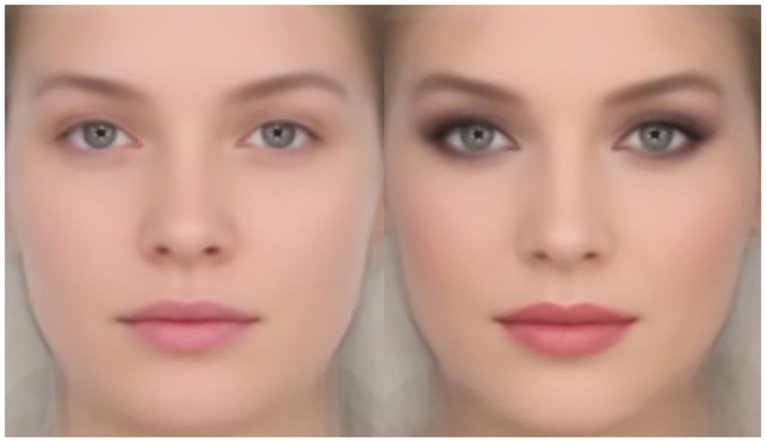
The average model without (left) and with cosmetics (right). These averages are cropped mid-forehead because several of the YouTube videos presented individuals in this way, resulting in insufficient information above this point for generating averages.

We captured a high-resolution screenshot of each model at the end of each video, where images of the models were presented before and after their application of cosmetics side-by-side. Models had a neutral expression and looked directly into the camera for the comparison. In addition, the two photographs were taken under the same lighting and camera conditions. From each comparison screenshot, we cropped the ‘before’ and ‘after’ versions of each model to produce two separate images. Final images were cropped just below the chin, at the hairline (or mid-forehead based on the limitations of the original), and tight to the widest part of the face (and so removing the ears). Given the variable nature of the images in terms of hairstyle, we chose models whose hair did not occlude their faces, and we masked loose hair in the lower portions of the images if it was not tied back. Images were resized to a height of 451 pixels. Given copyright restrictions, we present the average of models without cosmetics, and separately with cosmetics, in Fig 1 to illustrate. Averages were produced using JPsychomorph after landmarks were applied to the facial features in each image [32].

#### Procedure

Participants rated the attractiveness of the models using custom PsychoPy software [33]. Images were presented in a random order, and each participant rated each model only once, in a randomly selected cosmetics condition (i.e., either with or without cosmetics). This design was specifically chosen to prevent carryover effects between conditions [15,29]. Participants rated the attractiveness of the models on a 1 (*very unattractive*) to 7 (*very attractive*) scale, indicating their response via mouse click. Stimuli remained onscreen until a judgement was made.

A separate sample of participants judged the quantity of cosmetics worn by the models. These participants saw the ‘without’ and ‘with cosmetics’ images onscreen next to each other, and were asked ‘how much makeup has been applied to this face?’ Participants indicated their responses via mouse click on a 1 (*very light*) to 7 (*very heavy*) scale. Trials were presented in a random order. Though this is only a perceived measure of quantity, rather than an actual quantity of cosmetics, we believe it to be suitable as it is the perceived quantity that would affect the perceptions of observers. Importantly, other studies have found general agreement in the quantity of cosmetics applied by a professional makeup artist and the perceived amount of cosmetics being worn [31].

### Results

Each image was rated an average of 45 times (*SD* = 4.45). We examined agreement by calculating the pooled standard deviation for ratings in each cosmetics condition; without *SD*_p_ = 1.34; with cosmetics *SD*_p_ = 1.44. Responses were given on a 7-point scale, so the generally low variability indicates good agreement in ratings [15,34]. To examine effects of observer sex on ratings, the data were split by the sex of each observer before averaging. This resulted in four scores for each model—one in each cosmetics condition, as rated by men and women.

We also calculated the average amount of perceived cosmetics applied (*M* = 4.96, *SD* = 1.09), as judged by the separate sample of raters. These judgements of quantity were collected in order to be able to control for the varying amounts of cosmetics worn by each model in our analyses. However, this measure showed no relationship with the dependent variable (attractiveness) at all levels of observer sex and cosmetics, all *r*s < .25, *p*s > .160. As such, there was no reason to include quantity as a covariate, and we therefore analysed our results using a repeated measures ANOVA with model as the unit of analysis.

We focus here on the effect sizes of variables in order to estimate the real world effect of cosmetics on attractiveness. In particular, we utilise eta squared (*η*^**2**^**)** as a measure of effect size, which expresses how much each factor contributes to the total variance in attractiveness ratings as an interpretable percentage value, rather than partial eta squared, which does not sum across factors to one. We calculated *η*^**2**^ effect sizes for both main effects (Cosmetics, Observer Sex) and the interaction by dividing the sums of squares (SS) attributable to each effect by the total SS, calculated by summing the SS attributable to each effect and their respective errors. We also gave special consideration to the variance attributable to differences between items. This variation is typically ignored in repeated measures analyses since it usually represents variation between participants on the measured dependent variable, which is generally unimportant for repeated measures designs (which instead focus on variation *within* participants). However, in this case, it takes on a useful property. By using the images of the models as the unit of analysis, the variation between models represents variation in attractiveness arising due to the fact that models have different facial identities or appearances. We were therefore able to calculate an effect size for this ‘identity’ measure. The full results of the ANOVA are reported in Table 1, illustrating the effect sizes, their associated SS, and other statistics. It should be noted that there is no error term for conducting an *F* test on differences between models, and as such, no *F* ratio is calculated interactions with the Identity measure can be interpreted as an error term for that variable [35].

**Table 1:** Results of the analysis of variance from Study 1.

Source	*df*	*SS*	*η*^2^	*F*	*p*
Identity (I)	32	61.27	0.45		
Observer Sex	1	0.85	0.01	10.03	.003
Observer Sex × I	32	2.70	0.02		
Cosmetics	1	44.83	0.33	76.33	< .001
Cosmetics × I	32	18.79	0.14		
Observer Sex × Cosmetics	1	1.29	0.01	8.17	.007
Observer Sex × Cosmetics × I	32	5.05	0.04		
Total	131	134.78			

*Note*. *df* = degrees of freedom, *SS* = sums of squares.

Men assigned lower ratings of attractiveness (*M* = 3.74, 95% CI [3.47, 4.00]) than women (*M* = 3.89, [3.66, 4.13]), a result consistent with previous literature [15,36,37] which we do not pursue further here. Importantly, models were rated as more attractive with cosmetics (*M* = 4.39, [4.11, 4.68]) than without (*M* = 3.23, [2.95, 3.51]). The Observer Sex x Cosmetics interaction was driven by men rating faces without cosmetics as less attractive than women rating those same faces, *t*(32) = 4.32, *p* < .001, *d* = 0.75, but both sexes assigned similar ratings for models with cosmetics, *t*(32) = 0.42, *p* = .676, *d* = 0.07, indicating a larger influence of cosmetics on attractiveness for men. However, the effect size of this interaction was very small (*η*^2^ = 0.01), suggesting a relatively unimportant result.

**Fig 2:**
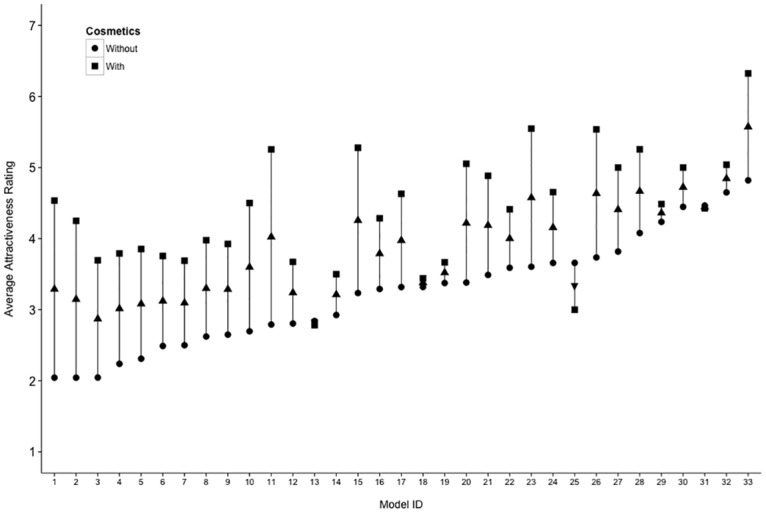
An illustration of the average attractiveness (combining ratings made by men and women) of each model, both without cosmetics and with cosmetics. Models are ordered in terms of increasing attractiveness without cosmetics. An upward pointing arrow indicates an increase in attractiveness with cosmetics, while a downward arrow indicates a decrease.

Of more importance was the Cosmetics x Identity interaction (*η*^2^ = 0.14), which indicates that the application of cosmetics altered the attractiveness of individual models differently. To examine this further, we computed a difference score for each model between their attractiveness with and without cosmetics, as rated by men and women. This difference illustrates the boost in attractiveness conferred by cosmetics, and we carried out a correlation between these values and the attractiveness of the models without cosmetics. Ratings assigned by both women and men showed a negative correlation between these values, *r*(31) = -.53, 95% CI [-.73, -.23], *p* = .001, and *r*(31) = -.48, [-.71, -.16], *p* = .005, respectively, indicating that the more attractive a model was, the less of an increase in attractiveness cosmetics conferred, a pattern which did not change when combining ratings given by men and women, *r*(31) = -.46, [-.69, -.14], *p* = .007 (see Fig 2).

Table 1 illustrates that the Identity effect size (*η*^2^ = 0.45) is 1.36 times larger than the effect size attributed to Cosmetics (*η*^2^ = 0.33). The differences in attractiveness between individuals explains more variance than an application of cosmetics, but the ratio of these effect sizes is much smaller than in previous accounts [15]. This suggests that a professional application of cosmetics (in comparison with self-application) is capable of producing a larger effect on attractiveness perceptions, although this remains smaller than the effect due to identity differences between women.

We conducted a final analysis to examine whether the cosmetics ‘look’ ascribed by the artist affected perceptions of attractiveness differently for men and women. The above analysis was repeated, but with the addition of ‘look’ as a source of variation between models. The three-way mixed model ANOVA revealed no significant main effects of cosmetics look or interactions with this factor, all *F*s < 1.18, *p*s > .320. However, it is worth noting that the ‘cosmetics look’ variable had low power (ranging from .076 to .242 across main effects and interactions), so further study is required to investigate the role of cosmetics look in perceived attractiveness.

## Study 2

The models used in Study 1 were women who had agreed to participate for the purposes of demonstration in a makeup tutorial. We have shown that the effect of cosmetics, when professionally-applied, results in a larger effect size compared with previous research [15]. Next, we investigate how cosmetics alter the attractiveness of a sample of women who are generally regarded as very attractive and earn a living based on their appearance—supermodels. We examine how much variation in attractiveness can be explained by cosmetics, and compare it with the effect size of identity, the differences in attractiveness between supermodels. Here, the effect size of identity should be smaller, given the potentially homogenous nature of the women in terms of attractiveness. How much of a benefit do cosmetics confer to highly attractive women, and in turn, do cosmetics overcome the differences in attractiveness between individuals?

### Method

#### Participants

One hundred new participants completed the study for course credit (age *M* = 19.28 years, *SD* = 1.46, 46 men), 14 of which were students at a Scottish university (age *M* = 19.28 years, *SD* = 1.68, one man), while the rest were students at a North American university (age *M* = 19.28 years, *SD* = 1.05, 45 men). A further sample of 14 North American students from the same university (age *M* = 20.50 years, *SD* = 1.28, 2 men) rated the quantity of cosmetics worn by the models.

The removal of the 14 participants from the Scottish university (who live in the UK rather than the US) did not change the pattern of results described below, aside from producing a significant main effect of Observer Sex, *F*(1, 44) = 18.64, *p* < .001, *η*^***2***^ = .02. As in Study 1, men provided lower ratings of attractiveness (*M* = 4.07, [3.91, 4.23]) than women (*M* = 4.32, [4.12, 4.53]). However, as this is a well-demonstrated effect and did not alter the presence of the interaction between cosmetics and observer sex, we include these extra participants for the additional validity they confer.

#### Ethics Statement

Ethical approval for all studies was obtained from the Gettysburg College institutional review board (IRB). All participants gave written informed consent before beginning the study. The Ethical Governance and Approval System at the University of Aberdeen granted approval for the study conducted there. Again, all participants gave written informed consent before beginning the study.

#### Stimuli

We collected images (*n* = 45) of supermodels without their makeup from the Internet. These images were casting photographs for Louis Vuitton’s Fall-Winter 2010 runway show. All pictures were taken with the models looking directly into the camera, with a neutral expression. We then collected images of the same women wearing cosmetics from professional photo shoots, and selected images where they had a neutral expression and were looking directly into the camera in order to match the casting photographs as closely as possible. However, these cosmetics photos were considerably less constrained in that the lighting varied between images, as did the amount of time between the two photos for each model. Therefore, while every care was taken to ensure similarity between these images and those of Study 1, we note that such limitations mean that any conclusions drawn from this study are necessarily more tentative.

**Fig 3:**
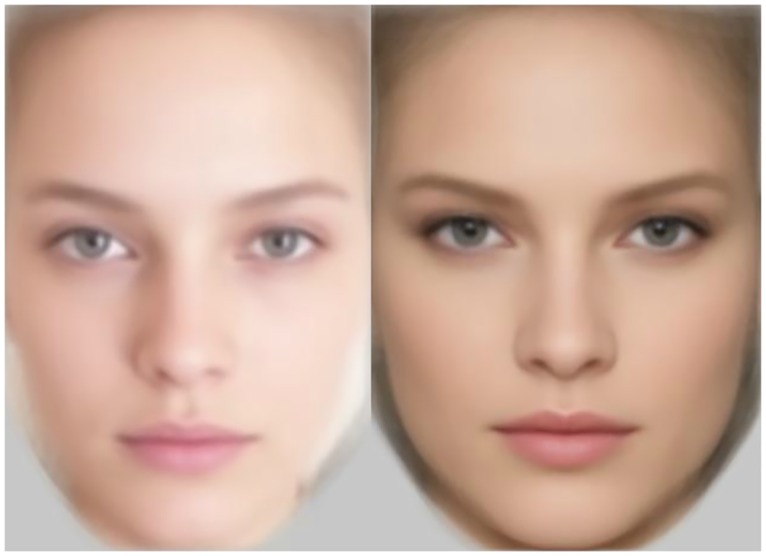
The average supermodel without (left) and with cosmetics (right).

Final images were cropped as in Study 1 to just below the chin, at the hairline, and tight to the widest part of the face (and so removing the ears). Hair was masked at the bottom of the images as before, and images were resized to a height of 250 pixels. Given copyright restrictions, we present the average of supermodels without cosmetics, and separately with cosmetics, in Fig 3 to illustrate.

#### Procedure

We used the same procedure as in Study 1. However, given that the photographs were of supermodels, there was a chance they would be recognised by observers. As such, we added a ‘recognise’ option onscreen where participants could indicate their recognition of the model rather than providing a rating of attractiveness. Familiarity with the models may result in unwanted influences on ratings. Across all images, an average of 4.95 trials were skipped (*SD* = 2.97). Ratings of attractiveness were therefore only collected for models that were not recognised by the raters.

#### Results

Each image received an average of 50 ratings (*SD* = 4.68). Agreement was calculated as before, using a pooled standard deviation for ratings within each cosmetics condition, and showed generally higher levels (lower variation) than Study 1; without cosmetics *SD*_p_ = 1.11; with cosmetics *SD*_p_ = 1.32. We split the data by the sex of the observer as before, and computed the average rating for each model in both cosmetics conditions as assigned by men and women.

We then averaged the ratings of quantity assigned by the separate sample of raters (*M* = 4.29, *SD* = 1.17) for use as a covariate in subsequent analyses. However, as in Study 1, the quantity measure showed no relationship with the dependent variable at any levels of each independent variable, all *r*s < .11, *p*s > .476. As such, analyses were carried out without inclusion of this covariate using repeated measures ANOVA, the results of which are summarised in Table 2. We also compared the perceived quantity ratings of the faces in Study 1 to the faces here, finding that the sample of supermodels (*M* = 4.29, [3.95, 4.63]) were perceived as wearing less cosmetics than the YouTube models (*M* = 4.96, [4.57, 5.36]), *t*(76) = 2.56, *p* = .012, *d* = 0.59.

**Table 2:** Results of the analysis of variance from Study 2.

Source	*df*	*SS*	*η*^2^	*F*	*p*
Identity (I)	44	58.08	0.43		
Observer Sex	1	0.37	0.00	2.79	.102
Observer Sex × I	44	5.85	0.04		
Cosmetics	1	33.35	0.25	47.89	< .001
Cosmetics × I	44	30.64	0.23		
Observer Sex × Cosmetics	1	1.73	0.01	20.66	< .001
Observer Sex × Cosmetics × I	44	3.68	0.03		
Total	179	133.70			

There is no error term for conducting an *F* test on differences between models, and as such, no ratio is calculated. *df* = degrees of freedom, *SS* = sums of squares. Interactions with the Identity measure can be interpreted as an error term for that variable [35].

As before, models were rated as more attractive with cosmetics (*M* = 4.53, [4.28, 4.77]) than without (*M* = 3.67, [3.49, 3.85]). The Observer Sex x Cosmetics interaction was again driven by men rating faces without cosmetics as less attractive than women rating those same faces, *t*(44) = 4.65, *p* < .001, *d* = 0.69, with both sexes perceiving the models as similarly attractive with cosmetics, *t*(44) = 1.37, *p* = .176, *d* = 0.21. However, as before, the effect size of this interaction was small (*η*^2^ = 0.01).

**Fig 4:**
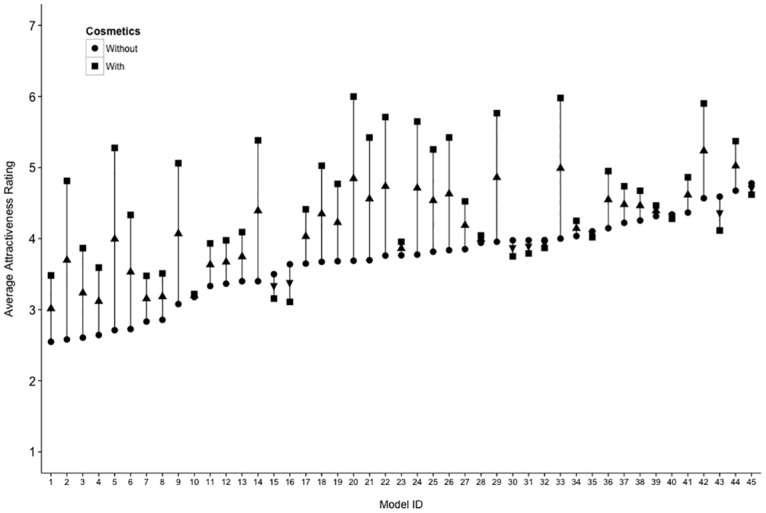
An illustration of the average attractiveness (combining ratings made by men and women) of each model, both without cosmetics and with cosmetics. Models are ordered in terms of increasing attractiveness without cosmetics. An upward pointing arrow indicates an increase in attractiveness with cosmetics, while a downward arrow indicates a decrease.

The effect size of the Cosmetics x Identity interaction (*η*^2^ = 0.23) was almost as large as the effect of cosmetics itself (*η*^2^ = 0.25), indicating the application of cosmetics affected the attractiveness of the supermodels differently. As before, we computed a difference score (for men and women’s ratings separately) between cosmetics conditions, and correlated this score with the attractiveness of the supermodels without cosmetics. Again, there was a negative correlation between the boost in attractiveness with cosmetics and the attractiveness of the models without cosmetics, for both women *r*(43) = -.40, [-.62, -.12], *p* = .006, and men *r*(43) = -.42, [-.64, -.14], *p* = .004, as well as when ratings given by both sexes were combined, *r*(43) = -.40, [-.62, -.12], *p* = .004. As before, this indicates that the more attractive the supermodel is perceived to be, the less of a boost in attractiveness cosmetics confer. This is illustrated in Fig 4.

The effect size of Identity (*η*^2^ = 0.43), due to differences in attractiveness between supermodels, was 1.72 times greater than that of cosmetics (*η*^2^ = 0.25), a ratio slightly larger than that observed in Study 1. Differences in attractiveness between individuals explained more variance than cosmetics, even among a sample of women regarded as highly attractive. The effect size of Cosmetics in this study was smaller than that found in Study 1, suggesting that cosmetics may produce a smaller increase in attractiveness for women who are already at the top end of the attractiveness scale, although the mean ratings for faces do not suggest a ceiling effect.

It is also worth noting that the effect size of Identity in this study was very close to the value reported in Study 1, which goes against our prediction that a sample of supermodels should have smaller between-person variability in attractiveness. However, this value is still notably smaller than the variation between individuals in a sample of university students [15], supporting our prediction of reduced between-person variability.

#### Combined Analyses

We have shown that professionally-applied cosmetics increase the attractiveness of both models and supermodels, with generally larger effect sizes than have been observed elsewhere [15]. Here, we combine the data from Study 1 with the data reported in previous work that provided an estimate of the effect size of cosmetics when self-applied to a student population [15]. This will allow a comparison of both model sets without and with cosmetics, and an overall comparison of the effect size of cosmetics and identity in a pooled setting of cosmetics use. We included only the models from Study 1 as these images were captured under more controlled conditions, similar to the images used in the previous work. In the initial study [15], there were 44 self-reported White women acting as models (age *M* = 21.18, *SD* = 1.94). Models applied their own cosmetics from a range of provided products, and were rated using the same procedure used here. See [15] for full details.

To conduct this analysis, we employed a three-way mixed ANOVA: Set (Students, YouTube) × Cosmetics (With, Without) × Observer Sex (Female, Male). Set represented a between-subjects factor, while the remaining factors were both within-subjects. As before, the model was the unit of analysis. Since a factorial ANOVA produces several statistical tests, we focus on the theoretically important outcomes. In this case, an interaction between Set and Cosmetics indicates that an application of cosmetics affects the model sets differently. We would predict models that received an application of professional cosmetics would appear more attractive.

As observed across the original data [15] and the two studies presented here, there was a main effect of Observer Sex, *F*(1, 75) = 122.45, *p* < .001, *η*^2^ = .04, following the usual pattern of men (*M* = 3.21, [3.05 3.37]) assigning lower ratings than women (*M* = 3.61, [3.44, 3.78]). Models also received higher ratings of attractiveness when viewed with cosmetics (*M* = 3.76, [3.58, 3.95]) compared to when they were viewed without (*M* = 3.05, [2.88, 3.23]), *F*(1, 75) = 97.35, *p* < .001, *η*^2^ = .12. Models from the YouTube set were also rated as more attractive (*M* = 3.82, [3.57, 4.06]) than those in the student set (*M* = 3.00, [2.79, 3.21]), *F*(1, 75) = 24.95, *p* < .001, *η*^2^ = .16.

The predicted interaction between Set and Cosmetics was present, *F*(1, 75) = 40.59, *p* < .001, *η*^2^ = .05. Bonferroni adjusted post-hoc tests revealed that without cosmetics, the YouTube models (*M* = 3.23, [2.97, 3.49]) were rated as slightly more attractive than models from the student set (*M* = 2.87, [2.65, 3.09]), *p* = .041, *d* = 0.24. However, with cosmetics, YouTube models (*M* = 4.39, [4.12, 4.67]) received significantly higher ratings of attractiveness than the student models (*M* = 3.12, [2.88, 3.37]), *p* < .001, *d* = 0.79, indicating a larger change in attractiveness with professionally-applied cosmetics than with self-applied cosmetics.

We can also draw comparisons between the sizes of our effects across all three studies (the two presented here and the student set). While *η*^2^ is ideal for comparing effect sizes within a study (the total always sums to 100%), comparison between studies is generally not recommended because the total variability depends on the study design and the number of independent variables [38]. However, the two studies reported here, as well as earlier data [15], use identical study designs, and the total variability is very similar in all cases (Study 1 *SS* total = 134.76, Study 2 *SS* total = 133.70, [15] *SS* total = 129.23). The main differences were the models used and the type of cosmetics applied. As such, we can justifiably make some comparisons between the effect sizes of cosmetics and identity across these studies.

While the effect size due to identity was similar in Studies 1 and 2 (*η*^2^ = .45 and *η*^2^ = .43, respectively), the earlier study using students showed a much larger effect (*η*^2^ = .69). The effect size of cosmetics in Studies 1 and 2 (*η*^2^ = .33 and *η*^2^ = .25, respectively), in contrast, were much larger than in the student study (*η*^2^ = .02). Therefore, while variation in attractiveness between individuals was somewhat greater among a sample of university students as compared to models and supermodels (as we would expect), the effect size of professionally-applied cosmetics was much larger than self-applied cosmetics. It is also important to note that the effect sizes obtained for the data in Study 2 are to be interpreted cautiously, given the more unconstrained nature of the images.

## General Discussion

Across several studies, we find that using cosmetics increases perceptions of attractiveness compared to no cosmetics, with several novel findings and caveats. First, we show that the effect size of cosmetics on attractiveness is large when those cosmetics have been professionally-applied, though the effect of identity is still greater. However, the difference between identity and cosmetics effects is much smaller than in a student sample of faces with self-applied cosmetics [15]. Second, we show that in a sample of supermodels with a smaller, more constrained effect size of identity (i.e., reduced between-person variance in attractiveness), identity is still more important than cosmetics, though the effect size of cosmetics is still larger than in previous cases. In both cases, but particularly the set of supermodels, we found evidence of an interaction between facial identity and cosmetics, indicating a differential effect of cosmetics on attractiveness. Further analysis revealed that the more attractive a face was without cosmetics, the less of an increase in attractiveness cosmetics conferred.

Across all studies, we observed that the effect of facial identity was larger than the effect of cosmetics. This finding extends previous research demonstrating that between-person variation is consistently larger than within-person manipulations of attractiveness [15,29]. Interestingly, the ratio between the effect sizes of identity and cosmetics in these studies (i.e., how much more variation identity explained than cosmetics in attractiveness judgements) is smaller than the comparison observed with emotional expression [29], suggesting that professionally-a—pplied cosmetics might be more effective at modulating attractiveness perceptions than facial expression, at least in female faces. Additionally, the finding that identity might be more important than within-person variation should perhaps be interpreted with caution. We refer to ‘identity’ in the current paper but use single, passport-style images of each model. However, individuals appear differently across different photographs, and this within-person variation in appearance has also been shown to affect perceived attractiveness [39].

A surprising source of variance in both studies was the interaction between identity and cosmetics. This finding, indicating that cosmetics affected different faces differently, was analysed further to reveal that the more attractive a face was initially, the less of an increase in attractiveness cosmetics conferred. While this is an intuitive finding, it has not been demonstrated before, and was particularly pronounced in the set of supermodels where the effect size of the interaction was almost as large as that of cosmetics itself. Cosmetics confer attractive patterns of colouration to faces, enhancing sex typical features in skin reflectance [18,20], as well as smoothing skin homogeneity and colour distribution [22,24,40]. Female faces that are considered attractive tend to have lighter skin, darker eyes, and redder lips than the average female face [41], which are all correlates of attractiveness [20,21], and in a recent study, are colourations that are conferred to faces by cosmetics [18]. It may be that the more attractive faces (i.e., of supermodels) already possess the most attractive features that cosmetics can alter, and so there is little change in attractiveness after an application. That less attractive faces receive more of an increase from cosmetics also has practical implications. By definition, the majority of women will lie around average attractiveness, and so a significant number of women could receive a boost in attractiveness from cosmetics.

We also found that the perceived quantity of cosmetics applied to faces played almost no role in the perceived attractiveness of faces with cosmetics. Recent evidence has shown that faces with lighter makeup are perceived as more attractive than faces with heavier makeup [42], which is at odds with our findings here. However, that study used different models for each cosmetics condition, conflating sources of cosmetics and identity variance, as well as using digitally applied cosmetics. While observers seem to find lighter cosmetics optimally attractive when given the choice to vary the quantity [16], no study as of yet has systematically shown that lighter cosmetics are optimally attractive for a given face. Our measurements here, as well as previous data [15], seem to suggest quantity does not play a large role in perceptions of attractiveness with cosmetics.

Combining image sets from previous research [15] with the findings from Study 1 revealed that, while the models from Study 1 were slightly more attractive than the models from the previous study, they were rated as significantly more attractive with cosmetics. After considering the similarity of designs and total variability across all studies (both here and in [15]), we compared the effect sizes of identity and cosmetics directly. Variability due to attractiveness between individuals (identity) was smaller among models and supermodels compared to university students, as predicted, but the effect size of cosmetics was noticeably larger for professionally-applied cosmetics. However, it is important to note that the sample sizes of models differed, and larger sample sizes might also result in greater between-person variability.

These findings have relevance for investigating the effects of cosmetics on social perceptions. There now exist estimates of the effect size of cosmetics when they are self-applied [15], and when they are applied professionally. In previous work [15], cosmetics explained just 2% of the variation in attractiveness, while the finding from a sample of models showed cosmetics explained 33% of the variation in attractiveness. This study demonstrated larger effect sizes of cosmetics when directly compared to previous research [15], though the studies used different sets of faces, and it is important to note that any effect size estimate calculated is ultimately based on the context of the research, and should be interpreted within this context [43]. However, the variances in the current and previous research are very similar, and the design of the studies is identical, meaning direct comparisons are valid and appropriate.

The literature examining the effect of cosmetics on social perceptions has, for the most part, used models with professionally-applied cosmetics in laboratory studies [9,10,17,30,31] as well as field experiments [12,13,44,45]. With our comparison of the effect size of cosmetics under both self-applied and professionally—aaaapplied conditions, it seems possible that some of the effects of cosmetics observed in the literature may be inflated. Further, women report higher self-confidence and engage in more social activities after a professional makeover [46] and this increase in self-confidence may translate into slight expression or postural differences in images, which could represent an additional within-person boost in attractiveness due to cosmetics.

There are some caveats to the study. Images were obtained from various Internet sources, and so were not as constrained in lighting or emotional expression as previous research [15]. Study 1 suffered less from this potential issue as images were collected from the same photographic session. As the images of supermodels with cosmetics were obtained from different sources, while the images of those women without cosmetics were obtained from the same source, the magnitude of the interaction between identity and cosmetics should be interpreted with caution. However, given its presence in Study 1 with more controlled stimuli, we think it safe to conclude that cosmetics affect more attractive individuals to a lesser extent than others. Furthermore, that such an effect was obtained in Study 2 with more variable photographs could be considered strong evidence. Since the images were more variable and cosmetics were confounded with variations in lighting (both considered noise in the current study), it seems likely an effect would be obtained under stricter conditions.

There now exists convincing evidence that alterations to within-person facial appearance via cosmetics, whether self-applied or professionally-applied, do not overcome between-person variability in attractiveness due to simple identity. Facial attractiveness is, to an extent, more about what you have, rather than what you do with it. However, we have uncovered here interesting caveats to this overarching and consistent finding. An increased skill level in applying cosmetics seems to offer a larger increase in attractiveness than self-applied cosmetics does—larger effects were clear when a professional makeup artist applied cosmetics. Furthermore, we have shown cosmetics affect faces of varying levels of attractiveness differently, particularly within a sample of faces with lower variation in attractiveness between individuals. More attractive individuals simply have less to gain from using cosmetics. These findings have theoretical implications for attractiveness research. Cosmetics is perhaps the most common form of modification of facial appearance, and we have shown that the currently reported literature, with its reliance on professionally-applied cosmetics, highlights an effect that does not seem achievable through everyday use.

How cosmetics affect attractiveness is a growing literature, and many studies use professionally-applied cosmetics as a means to examine this change. We have shown that professionally-applied cosmetics seem to explain a larger proportion of variation in attractiveness judgements than self-applied cosmetics, a category which the vast majority of cosmetics users fall under. This could suggest an inflation of the effect of cosmetics in the current literature, with cosmetics increasing attractiveness beyond what is achievable through everyday means. Additionally, we have illustrated that cosmetics affect women differently—more attractive women, particularly supermodels, gain less of a boost in attractiveness from cosmetics than do less attractive women. Importantly, the effect size of identity, or between-person variance in attractiveness, was larger than the effect of cosmetics in both studies. We conclude that, when it comes to cosmetics, individual differences in facial appearance are ultimately more important than even a professional application of cosmetics.

## Supporting Information

S1 Dataset Data from Study 1. Each participant rated all 33 YouTube models, but each model appeared in a randomly selected cosmetics condition. All conditions are stated in the data. We averaged across participants for each image, building a score for each identity under both cosmetics conditions.
(XLS)

S2 Dataset Data from Study 2. Each participant rated all 45 supermodels, but each model appeared in a randomly selected cosmetics condition. All conditions are stated in the data. We averaged across participants for each image, building a score for each identity under both cosmetics conditions.
(XLS)

S3 Dataset Data from the quantity raters in both studies. Sheet 1 contains the quantity data from Study 1, and Sheet 2 contains the quantity data for Study 2. Participants compared each model without and with cosmetics, indicating how much cosmetics the faces were wearing.
(XLS)
